# Evaluation of Metformin on Cognitive Improvement in Patients With Non-dementia Vascular Cognitive Impairment and Abnormal Glucose Metabolism

**DOI:** 10.3389/fnagi.2018.00227

**Published:** 2018-07-27

**Authors:** Yufeng Lin, Kaiyuan Wang, Chunchao Ma, Xuesong Wang, Zhongying Gong, Rui Zhang, Dawei Zang, Yan Cheng

**Affiliations:** ^1^Department of Neurology, Tianjin Medical University General Hospital, Tianjin, China; ^2^Department of Neurology, Tianjin First Center Clinical College of Tianjin Medical University, Tianjin, China; ^3^Department of Anesthesiology, Tianjin Medical University Cancer Institute and Hospital, Tianjin, China

**Keywords:** non-dementia vascular cognitive impairment, glucose metabolism disorders, insulin resistance, metformin, carotid intima–media thickness

## Abstract

**Objective:** Recent studies have suggested that metformin can penetrate the blood–brain barrier, protecting neurons *via* anti-inflammatory action and improvement of brain energy metabolism. In this study, we aim to investigate the effect of metformin on cognitive function in patients with abnormal glucose metabolism and non-dementia vascular cognitive impairment (NDVCI).

**Methods:** One hundred patients with NDVCI and abnormal glucose metabolism were randomly allocated into two groups: metformin and donepezil (*n* = 50) or acarbose and donepezil (*n* = 50). The neuropsychological status, glucose metabolism, and common carotid arteries intima–media thickness (CCA-IMT) before and after a year of treatment, were measured and compared between the groups.

**Results:** Ninety four patients completed all the assessment and follow-up. After a year of treatment, there was a decrease in Alzheimer’s disease Assessment Scale-Cognitive Subscale scores and the duration of the Trail Making Test in the metformin-donepezil group. Furthermore, these patients showed a significant increase in World Health Organization–University of California–Los Angeles Auditory Verbal Learning Test scores after treatment (all *P* < 0.05). However, there was no obvious improvement in cognitive function in the acarbose-donepezil group. We also observed a significant decrease in the level of fasting insulin and insulin resistance (IR) index in the metformin-donepezil group, with a lower CCA-IMT value than that in the acarbose-donepezil group after a year of treatment (*P* < 0.05).

**Conclusion:** We conclude that metformin can improve cognitive function in patients with NDVCI and abnormal glucose metabolism, especially in terms of performance function. Improved cognitive function may be related to improvement of IR and the attenuated progression of IMT.

**Trial Registration:** ChiCTR-IPR-17011855.

## Introduction

Vascular cognitive impairment (VCI) refers to a syndrome in which there is evidence of clinical stroke or subclinical vascular brain damage, with damage to at least one domain of cognitive function (Gorelick et al., 2011; Platt and Riedel, 2011; Umemura et al., 2011; Sachdev et al., 2014). VCI is caused by dysfunction of neurovascular units and blood flow regulation disorders, of which oxidative stress in vascular endothelial cells and glial cells, and neuronal injury caused by immune inflammatory responses are key factors (Gorelick and Nyenhuis, 2013). Non-dementia vascular cognitive impairment (NDVCI) and vascular dementia (VD) are two stages of VCI that can involve various cognitive domains, especially in NDVCI (Sun et al., 2015). NDVCI is often overlooked by patients and clinicians because the patient’s daily abilities are not affected. However, about 50% of patients with NDVCI will progress to dementia within 5 years (Sutton et al., 2012). Therefore, the early diagnosis and intervention of NDVCI is becoming the focus of worldwide stroke research and therapy.

Glucose metabolism disorder, insulin resistance (IR), and atherosclerosis are closely related to VCI, and dysfunctional glucose metabolism plays a role in the destruction of the insulin receptor system (Luchsinger, 2012). Metformin, a biguanide hypoglycemic drug, is the most widely used oral hypoglycemic agent. It mainly acts by improving insulin sensitivity and promoting glucose uptake while decreasing hepatic glycogen synthesis (He et al., 2009; Labuzek et al., 2010; Foretz and Viollet, 2014). Recent studies have shown that metformin can rapidly penetrate the blood–brain barrier to protect neurons through anti-inflammatory processes and improvement of brain energy metabolism (Ying et al., 2014). Meanwhile, neuroimaging like magnetic resonance imaging (MRI) and positron emission tomography have already proved that the neuroinflammation and brain metabolic stress may damage cognitive function (Lee et al., 2010; De Felice and Lourenco, 2015). Thus, it is hypothesized that metformin treatment could improve the cognitive impairment.

As the alpha-glucosidase inhibitor, acarbose is another commonly applied drug for monotherapy of hyperglycemia with favorable efficacy and high degree of safety (Rosak and Mertes, 2012). Therefore, in the present study, we used acarbose as a control therapy, and to investigate whether metformin or acarbose in combination with donepezil can improve cognitive function in patients with NDVCI with impaired glucose metabolism.

## Materials and Methods

### Participants

The study was approved by the ethics committee of Tianjin First Center Hospital and all participants gave written informed consent in accordance with the Declaration of Helsinki before participation. The study was designed as a randomized controlled clinical trial with related registration No. ChiCTR-IPR-17011855. We recruited 100 patients with NDVCI and impaired glucose metabolism (male, *n* = 58; female, *n* = 42; and mean age 68.1 ± 7.8 years) from the Department of Neurology, Tianjin First Center Hospital.

Diagnosis of NDVCI was based on the presence of vascular diseases of the central nervous system and cognitive dysfunction. Inclusion criteria were: (1) confirmed decline in cognitive function reported during an interview with the patient, their caregiver, or a family member; (2) neural imaging (CT or MRI) showing multiple lacunar infarction and/or extensive white matter lesions; (3) cognitive dysfunction grade = 0.5; (4) Mini-Mental State Examination score ≥ 20; (5) Montreal Cognitive Assessment score < 26; (6) activities of daily living (ADL) score ≤ 26; and (7) abnormal glucose metabolism initially confirmed with an oral glucose tolerance test and insulin release test. Exclusion criteria included any patients with: (1) severe aphasia or disturbance of consciousness preventing communication; (2) tumor, heart, lung, or other organ failure; (3) a long history of alcohol abuse; (4) mental illness; (5) cognitive impairment caused by other factors; or (6) dementia. Enrolled patients with severe drug-related side effect, incomplete follow-up data, or refusal of neuropsychological assessment will also be excluded during the observation.

### Treatment Regimens

We randomly allocated 100 patients into two groups. Patients in these groups underwent different treatments: metformin or acarbose (*n* = 50 per group). Participants allocated to the metformin group were administered metformin 500 mg t.i.d. and donepezil 10 mg q.n., while those allocated to the acarbose group were administered acarbose 50 mg t.i.d. and donepezil 10 mg q.n. Side effects including gastrointestinal adverse reactions were closely observed and treated immediately. Physicians in charge of cognitive function assessment and clinical data collecting were blinded to the therapeutic protocol.

### Laboratory Tests

Venous blood was collected from all patients admitted to the hospital after fasting for 8 h in the morning. Bloods were centrifuged within 1 h. We detected levels of blood glucose, total triglyceride, low density lipoprotein, high density lipoprotein, homocysteine, and hemoglobin A1c (HbA1c) with an OLYMPUS AU-400 automatic biochemical analyzer. After a year of treatment, levels of blood glucose and HbA1c were detected and recorded with the same method while the fasting insulin level was detected with radioimmunoassay. The diagnostic criteria of IR was defined as IR index = fasting insulin (mU/L)^∗^fasting blood glucose (mmol/L)/22.5, where IR exists if the value >2.8 (McLaughlin et al., 2014).

### Intima–Media Thickness Measurement

All patients underwent carotid vascular ultrasound examination within 72 h of admission and a year after treatment to measure the common carotid arteries intima–media thickness (CCA-IMT). An IMT ≥1.0 mm indicated the existence of carotid atherosclerosis.

### Assessment of Cognitive Function

Cognitive function of all participants was assessed before and after a year of treatment with the following scales: (1) the Alzheimer’s Disease Assessment Scale-Cognitive Subscale (ADAS-Cog) was used to evaluate total cognitive function (Bagnoli et al., 2012); (2) the World Health Organization–University of California–Los Angeles Auditory Verbal Learning Test (WHO–UCLA AVLT) provided measures of memory ability (Maj et al., 1994); and (3) the Trail Making Test (TMT) was used to assess executive function, with part A reflecting speed of perceptual motion, and part B comprising not only the former but also reflecting the ability to perform concept and attention switching (O’Sullivan et al., 2005). All assessments were conducted by the same attending physician in a quiet environment between 4 and 6 p.m.

### Statistical Analysis

To detect a 25% difference in ADAS-Cog score 1 year after treatment (mean: 16.3 with standard deviation: 4.6; data from our preliminary observation) between the acarbose group and metformin group at a ratio of 1:1 with a test power of 0.9 and at a significance level of 0.05, at least 27 participants would be required in each group. All statistical analyses were performed with SPSS 17.0 (SPSS, Inc., Chicago, IL, United States). Normality of distributions was assessed by the Shapiro–Wilks test, and the data with *P*-value >0.05 were determined as normal distribution. Continuous data with normal distribution are expressed as mean ± standard deviation and were compared with a two-sample *t*-test or paired *t*-test, as appropriate. Categorical variables were tested with the Chi-square test. *P*-values <0.05 were considered to be statistically significant.

## Results

### Baseline Characteristics

**FIGURE 1:**
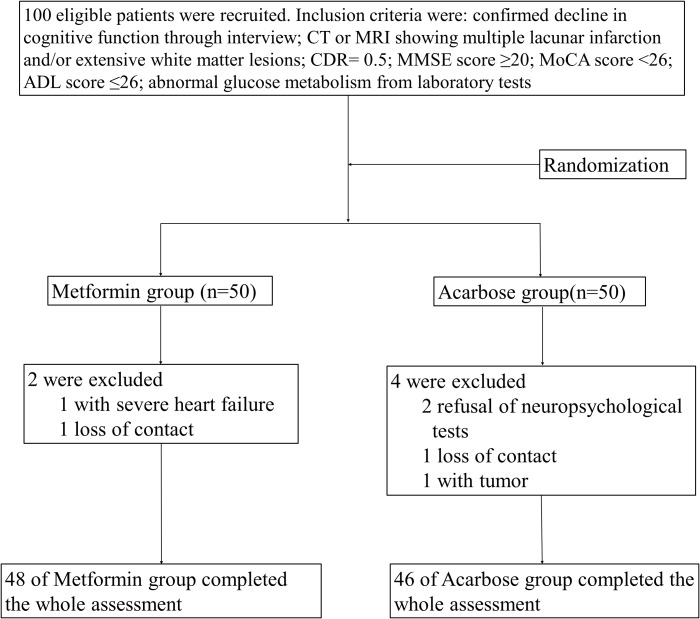
Flow chart of enrollment, allocation, follow-up, and analysis. CDR, cognitive dysfunction grade; MMSE, Mini-Mental State Examination; MoCA, Montreal Cognitive Assessment; ADL, activities of daily living.

Six of the 100 patients failed to complete the after-treatment assessments due to severe cardiac failure, refusal, or loss of contact. A total of 94 patients, i.e., 48 patients from the metformin group and 46 patients from the acarbose group, underwent all the neuropsychological tests (**Figure 1**). Data from all the observed indicators of these patients complied with normal distribution and were further analyzed. There were no significant differences in age, sex, glucose metabolism, body mass index, mean blood pressure, duration of abnormal glucose metabolism, total triglyceride, low density lipoprotein, high density lipoprotein, or homocysteine between the two groups on hospital admission (all *P* > 0.05; **Table 1**).

**Table 1:** Demographics and clinical data of two groups.

	Metformin group (*n* = 48)	Acarbose group (*n* = 46)	*P*
Age (years)	66.5 ± 7.6	67.4 ± 8.2	0.582^a^
Male (*n*/%)	26 (54%)	27 (59%)	0.658^b^
Body mass index (kg/m^2^)	24.7 ± 3.3	25.4 ± 3.5	0.321^a^
Mean blood pressure (mmHg)	99.9 ± 10.8	103.2 ± 12.1	0.167^a^
Duration of abnormal glucose metabolism (years)	15 ± 5.6	14 ± 4.9	0.360^a^
Total triglyceride (mmol/L)	5.34 ± 1.47	5.53 ± 1.39	0.522^a^
Low density lipoprotein (mmol/L)	2.83 ± 0.72	2.79 ± 0.67	0.781^a^
High density lipoprotein (mmol/L)	1.68 ± 0.43	1.59 ± 0.38	0.286^a^
Homocysteine (μmol/L)	14.09 ± 2.87	14.73 ± 3.01	0.294^a^


*^a^Two-samplet-test. ^b^Chi-square test.*

### Cognitive Function

The results of neuropsychological tests are shown in **Table 2**. Before treatment there were no significant differences in ADAS-Cog or WHO–UCLA AVLT scores, or TMT times between the two groups (all *P* > 0.05). After a year of treatment, there were significant decreases in ADAS-Cog scores and TMT times and increases in WHO–UCLA AVLT scores from baseline in the metformin group (all *P* < 0.05). In contrast, there were no significant changes in ADAS-Cog or WHO–UCLA AVLT scores, or TMT times after treatment in the acarbose group (all *P* > 0.05). After a year of treatment, ADAS-Cog scores and TMT times were lower, while WHO–UCLA AVLT scores were higher in patients treated with metformin than in those treated with acarbose (all *P* < 0.05).

**Table 2:** Comparison of neuropsychological tests before and after treatment between two groups (*
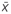
*±*s*).

	Group	*n*	Before treatment	One year after treatment	*P*
ADAS-Cog score	Metformin	48	16.9 ± 6.1	11.9 ± 3.7	<0.001^b^
	Acarbose	46	17.0 ± 5.9	15.9 ± 3.9	0.295^b^
	*P*		0.936^a^	<0.001^a^	
WHO–UCLA AVLT score	Metformin	48	16.1 ± 4.1	17.9 ± 4.5	0.043^b^
	Acarbose	46	15.9 ± 3.9	15.6 ± 4.3	0.727^b^
	*P*		0.809^a^	0.013^a^	
TMT-A (s)	Metformin	48	40.3 ± 16.1	34.6 ± 11.1	0.046^b^
	Acarbose	46	39.8 ± 16.3	41.2 ± 17.3	0.690^b^
	*P*		0.881^a^	0.029^a^	
TMT-B (s)	Metformin	48	95.8 ± 25.2	86.1 ± 22.0	0.047^b^
	Acarbose	46	96.7 ± 26.4	98.2 ± 19.6	0.856^b^
	*P*		0.866^a^	0.006^a^	


*ADAS-Cog score, Alzheimer’s Disease Assessment Scale-Cognitive Subscale (ADAS-Cog); WHO–UCLA AVLT score, the World Health Organization–University of California–Los Angeles Auditory Verbal Learning Test; TMT, trail making test.^a^Two-samplet-test. ^b^Pairedt-test.*

### Glucose Metabolism

There were no significant differences in levels of fasting blood glucose, fasting insulin, IR index, or HbA1c between the two groups before treatment (*P* > 0.05; **Table 3**). After a year of treatment, fasting insulin levels and IR index in the metformin group were significantly decreased (*P* < 0.05), but differences in fasting blood glucose and HbA1c levels were not statistically different (*P* > 0.05).

**Table 3:** Comparison of glucose metabolism parameters before and after treatment between two groups (*
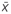
*±*s*).

	Group	*n*	Before treatment	One year after treatment	*P*
Fasting blood glucose (mmol/L)	Metformin	48	7.8 ± 2.2	7.6 ± 2.4	0.671^b^
	Acarbose	46	8.1 ± 2.6	7.9 ± 2.7	0.718^b^
	*P*		0.547^a^	0.570^a^	
Fasting insulin (mU/L)	Metformin	48	14.8 ± 5.6	11.9 ± 5.7	0.014^b^
	Acarbose	46	14.5 ± 5.7	17.1 ± 5.1	0.023^b^
	*P*		0.797^a^	<0.001^a^	
IR index	Metformin	48	4.7 ± 1.3	4.0 ± 1.1	0.005^b^
	Acarbose	46	4.4 ± 1.9	5.0 ± 2.9	0.243^b^
	*P*		0.372^a^	0.028^a^	
HbA1c (%)	Metformin	48	6.9 ± 1.4	6.7 ± 1.2	0.454^b^
	Acarbose	46	6.6 ± 1.2	6.8 ± 1.7	0.516^b^
	*P*		0.268^a^	0.742^a^	


*IR index, insulin resistance index; HbA1c, hemoglobin A1c. ^a^Two-samplet-test.^b^Pairedt-test.*

In patients treated with acarbose, fasting insulin levels were elevated after a year of treatment (*P* = 0.023), while fasting blood glucose levels, IR index, and HbA1c levels did not change significantly (*P* > 0.05).

Compared with the acarbose group, the fasting insulin levels and IR index were significantly decreased in the metformin group (*P* < 0.05), but there were no significant differences in fasting blood glucose or HbA1c levels (*P* > 0.05).

### Intima–Media Thickness

The results showed that there were no significant differences in CCA-IMT between the two groups at admission (*P* > 0.05). After a year of treatment, there was no significant change in the CCA-IMT of the metformin group (*P* > 0.05), while there was a significant increase in the CCA-IMT of the acarbose group (*P* < 0.05). Furthermore, the CCA-IMT value for the metformin group was significantly lower than that of the acarbose group (*P* < 0.05; **Table 4**).

**Table 4:** Comparison of the common carotid arteries intima–media thickness between two groups (*
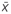
*±*s*, mm).

Group	*n*	Before treatment	One year after treatment	*P*
Metformin	48	0.90 ± 0.20	0.91 ± 0.16	0.787^b^
Acarbose	46	0.91 ± 0.15	0.98 ± 0.17	0.039^b^
*P*		0.785^a^	0.043^a^	


*^a^Two-samplet-test.^b^Pairedt-test.*

## Discussion

With the current increase in the aging population, the incidence of cerebrovascular disease-induced VCI and surgery related cognitive dysfunction is also increasing (Wang et al., 2017). VCI includes NDVCI and VD (Turan et al., 2010). Research showed that half of the patients with NDVCI will develop into dementia within 5 years (Sutton et al., 2012). Therefore, investigating potential interventions for NDVCI are extremely important and could provide the basis for clinical prevention of VD. Unlike neurodegenerative dementia, cognitive impairment caused by cerebrovascular disease can be prevented and treated, especially through control of risk factors, such as anti-platelet aggregation, high blood pressure, and abnormal blood glucose and blood lipid levels. In addition, recent research indicates that dysfunction in the cerebral acetylcholine pathway leads to diminished acetylcholine levels in patients with VCI, providing neurobiological evidence for therapy with cholinergic enzyme inhibitors (Platt and Riedel, 2011). Indeed, donepezil can improve cognitive impairment in patients with mild to moderate VCI, and also improves behavioral symptoms and ADL (Roman et al., 2010). Therefore, in this study, we used donepezil as the main treatment for impaired cognitive function of patients with VCI.

Previous studies have shown that glucose metabolism can lead to varying degrees of cognitive dysfunction (Banks et al., 2012). The mechanisms underlying this association are complex and generally considered to be the result of multiple factors, including mitochondrial dysfunction, oxidative stress, protein glycation, increased aldose reductase activity, and intracellular signal transduction pathway abnormalities causing impaired neuronal function (Craft and Watson, 2004; Kodl and Seaquist, 2008). In addition, IR is also an important risk factor for cognitive decline, mainly *via* its association with hyperinsulinemia, and glucose and lipid metabolism disorders. Abnormal glucose and lipid metabolism can induce vascular disease, resulting in reduced cerebral blood flow. This can create obstacles for the ability of the brain to enable understanding, processing, and integration of information, leading to learning and memory impairments (Umemura et al., 2011; Micha et al., 2017; Song and Kim, 2017). Additionally, IR can reduce the expression of insulin-like growth factor 1 and decrease choline acetyltransferase activity in neurons, thus impeding the release of acetylcholine and leading to decreased cognitive function. Animal experiments have confirmed that rats with IR have increased advanced glycation end products and increased expression of their receptors, as well as decreased peroxisome receptor γ (PPARγ) expression in the cerebral cortex and hippocampus. The combination of advanced glycation end products and corresponding receptors directly or indirectly leads to the occurrence and development of cerebrovascular disease, whereas reduced PPARγ expression can exacerbate this central nervous injury. Therefore, for patients with VCI and abnormal glucose metabolism, improvement of accompanying IR may have a protective effect on neurons.

Metformin is a first-line hypoglycemic drug approved by the US Food and Drug Administration. Therefore, the effects of metformin on diabetes-related cognitive dysfunction have received widespread concern in recent years. Many studies have demonstrated that metformin has anti-inflammatory and antioxidant physiological effects, in addition to improving IR (Martin-Montalvo et al., 2013; Dai et al., 2014; Shafaee et al., 2015). Compared with other hypoglycemic agents, metformin has been shown to effectively improve cognitive function in patients with diabetes mellitus in terms of language learning and execution ability (Herath et al., 2016). However, the effect of metformin on VCI has not previously been reported. In this study, we compared the use of donepezil combined with metformin or acarbose in the treatment of NDVCI with glucose metabolism. The results showed that there were significant reductions in ADAS-Cog scores following a year of treatment with donepezil plus metformin compared with donepezil acarbose. Furthermore, WHO–UCLA AVLT scores were increased and TMT times were significantly shorter following treatment with metformin compared to acarbose. These results suggest that cognitive function in patients treated with metformin improved significantly, with more obvious improvements in performance ability than in memory function. We also observed improved glucose metabolism in the metformin group with reduced fasting insulin levels and IR index after a year of treatment.

In this study, the observed effect of metformin on NDVCI may result from several mechanisms. Metformin could activate AMP-activated protein kinase (AMPK) (He et al., 2009), enhancing activity in the AMPK-Sirtuin 1 (SIRT1)-peroxisome coactivator 1α (PGC1α) pathway (Qin et al., 2006; Mayoral et al., 2015), and further activating PPARγ. This would strengthen central cholinergic acetylation enzyme activity and increase insulin-like growth factor-1 expression, leading to the maintenance of cholinergic nerve function and attenuating cognitive decline as seen in rats with cognitive dysfunction (Mayoral et al., 2015). Similarly, Gao et al. (2010) and Hildreth et al. (2015) found that pioglitazone can also improve the sensitivity of insulin by activating the PPARγ pathway, thereby improving cognitive function in patients with VCI. In addition, as a longevity gene, SIRT1 could also be activated by metformin to enhance plasticity of the central nervous system, and thus improve cognitive learning abilities (Gao et al., 2010).

The common carotid artery is a typical medium-sized artery that can be easily detected on the body surface with ultrasound. The severity of lesions in this artery can indirectly reflect the atherosclerotic status of the intracranial and coronary arteries. IMT is a valuable indicator of early atherosclerosis and can predict adverse cardiovascular and cerebrovascular events (Li et al., 2017). In this study, we found that the progression of CCA-IMT in patients treated with metformin was significantly less than that of patients treated with acarbose at 1 year. This may further contribute to increased blood supply to brain tissue and improved cognitive function. Previous studies have shown that metformin can slow the progress of abnormal IMT, potentially acting to increase antioxidant protection, resulting in reductions in both oxidative damage accumulation and chronic inflammation (Martin-Montalvo et al., 2013). In the present study, the protective role of metformin on the carotid artery may be a further mechanism by which it improves cognitive function in VCI.

Several limitations should be noted in this study. First, due to the small sample size and lack of one control group without antidiabetics, our results can only inform preliminary conclusions, which need to be further confirmed by larger multi-center clinical trials with larger sample sizes. Second, the observation period of this study was 1 year. The ameliorative effects of metformin on long-term cognitive function and its therapeutic role in abnormal glucose metabolism for patients with NDVCI should also be evaluated in studies with longer follow-up. Finally, the present study found that metformin had different effects on different aspects of cognitive function, and that improvements in patients’ performance was superior to their memory performance. Further research is required to understand the mechanisms underlying these patterns of improvement.

In summary, impaired cognitive function in patients with NDVCI and abnormal glucose metabolism is closely related to IR. Treatment that combines metformin with donepezil can improve IR and reduce fasting insulin levels in such patients. Meanwhile, metformin could also attenuate carotid artery lesions, contributing to the improved cognitive function in patients with NDVCI.

## Author Contributions

YC and DZ designed the study protocol and revised the manuscript. YL, CM, XW, and ZG performed the observations. YL and KW wrote the manuscript. KW and RZ analyzed the collected data.

## Conflict of Interest Statement

The authors declare that the research was conducted in the absence of any commercial or financial relationships that could be construed as a potential conflict of interest.
