# Corrigendum: Evaluation of Metformin on Cognitive Improvement in Patients With Non-dementia Vascular Cognitive Impairment and Abnormal Glucose Metabolism

**DOI:** 10.3389/fnagi.2018.00322

**Published:** 2018-10-12

**Authors:** Yufeng Lin, Kaiyuan Wang, Chunchao Ma, Xuesong Wang, Zhongying Gong, Rui Zhang, Dawei Zang, Yan Cheng

**Affiliations:** ^1^Department of Neurology, Tianjin Medical University General Hospital, Tianjin, China; ^2^Department of Neurology, Tianjin First Center Clinical College of Tianjin Medical University, Tianjin, China; ^3^Department of Anesthesiology, Tianjin Medical University Cancer Institute and Hospital, Tianjin, China

**Keywords:** non-dementia vascular cognitive impairment, glucose metabolism disorders, insulin resistance, metformin, carotid intima–media thickness

In the original article, we missed an affiliation for Yufeng Lin. The new affiliations have been added and reordered accordingly.

The authors apologize for this error and state that this does not change the scientific conclusions of the article in any way.

The original article has been updated.

## Conflict of interest statement

The authors declare that the research was conducted in the absence of any commercial or financial relationships that could be construed as a potential conflict of interest.

